# Single-Molecule Imaging Reveals Differential AT1R Stoichiometry Change in Biased Signaling

**DOI:** 10.3390/ijms25010374

**Published:** 2023-12-27

**Authors:** Gege Qin, Jiachao Xu, Yuxin Liang, Xiaohong Fang

**Affiliations:** 1 Key Laboratory of Molecular Nanostructure and Nanotechnology, CAS Research/Education Center for Excellence in Molecular Sciences, Institute of Chemistry, Chinese Academy of Sciences, Beijing 100190, China; 2 University of Chinese Academy of Sciences, Beijing 100049, China; 3 Key Laboratory of Molecular Developmental Biology, Institute of Genetics and Developmental Biology, Chinese Academy of Sciences, Beijing 100101, China; 4 Hangzhou Institute of Medicine (HIM), Chinese Academy of Sciences, Hangzhou 310022, China

**Keywords:** single-molecule imaging, angiotensin II type 1 receptor, biased activation, stoichiometry

## Abstract

G protein-coupled receptors (GPCRs) represent promising therapeutic targets due to their involvement in numerous physiological processes mediated by downstream G protein- and β-arrestin-mediated signal transduction cascades. Although the precise control of GPCR signaling pathways is therapeutically valuable, the molecular details for governing biased GPCR signaling remain elusive. The Angiotensin II type 1 receptor (AT1R), a prototypical class A GPCR with profound implications for cardiovascular functions, has become a focal point for biased ligand-based clinical interventions. Herein, we used single-molecule live-cell imaging techniques to evaluate the changes in stoichiometry and dynamics of AT1R with distinct biased ligand stimulations in real time. It was revealed that AT1R existed predominantly in monomers and dimers and underwent oligomerization upon ligand stimulation. Notably, β-arrestin-biased ligands induced the formation of higher-order aggregates, resulting in a slower diffusion profile for AT1R compared to G protein-biased ligands. Furthermore, we demonstrated that the augmented aggregation of AT1R, triggered by activation from each biased ligand, was completely abrogated in β-arrestin knockout cells. These findings furnish novel insights into the intricate relationship between GPCR aggregation states and biased signaling, underscoring the pivotal role of molecular behaviors in guiding the development of selective therapeutic agents.

## 1. Introduction

G protein-coupled receptors (GPCRs) constitute the largest family of membrane-signaling receptors [1]. Given their pivotal role in regulating nearly all physiological processes within the human body, GPCRs serve as the target for a substantial portion, estimated to be at least 30–40%, of all approved drugs [2]. The diverse functionality of numerous GPCRs is intricately linked to their downstream signaling pathways, encompassing both G protein-mediated signaling and β-arrestin-mediated signaling [3,4]. Biased ligands that selectively modulate these two pathways, varying in their signaling efficacy, have emerged as promising candidates with significant therapeutic potential. They offer a compelling approach to drug design, emphasizing the enhancement of signaling pathways responsible for therapeutic benefits while minimizing those associated with undesirable side effects [5,6]. Consequently, an enhanced molecular comprehension of biased signaling holds scientific significance and clinical relevance.

Despite considerable efforts in comprehending the biased signaling of GPCRs controlled by ligands [7,8,9], the underlying physiological molecular mechanisms governing function-selective GPCR signaling have only recently begun to be unraveled [10]. Live-cell single-molecule fluorescence imaging analysis is an emerging technique to study stoichiometry and diffusion of GPCRs in biased signaling. Through the introduction of the inhibitor, single-molecule imaging results showed that the inhibition of G protein signaling decreased the oligomerization of C-X-C chemokine receptor type 4 (CXCR4) in its signal transduction process [11]. Recent triple-color single-molecule imaging confirmed similar findings, indicating that formyl peptide receptor 1 (FPR1) oligomerization corresponded to prolonged FPR1-G protein interaction and was increased upon full agonist stimulation [12]. The platelet-activating factor receptor (PAFR) has also been confirmed to preferentially propagate G protein signaling in the form of oligomers, as opposed to its role as a monomeric promoter when activating the β-arrestin pathway. Intriguingly, β-arrestin prevented PAFR oligomer formation [13]. Conversely, through SNAP-tag technology, it was found that full agonist stimulation had no effect on the aggregation state of the β_1_-Adrenergic receptor (β_1_-AR) and the γ-aminobutyric acid type B receptor (GABA_B_) [14]. Furthermore, the enhanced oligomerization of β_2_-AR occurred after the β-arrestin-biased ligand stimulation, and this oligomerization was β-arrestin-dependent [15]. Combining single-molecule imaging and bioluminescence resonance energy transfer (BRET) analyses, the dimerization of µ-opioid receptor (µOR) was also related to β-arrestin recruitment [16]. The accumulation of evidence highlights the relevance of the GPCR aggregation state to biased signal transduction, while the existing controversy suggests that many molecular knowledge gaps remain in the mechanism that tunes the bias of GPCR signaling.

The Angiotensin II type 1 receptor (AT1R) also belongs to the class A GPCR family [17]. AT1R dysregulation is intricately involved in pathologies such as atherosclerosis, hypertension, and heart failure, rendering it a primary target for most current cardiovascular pharmacotherapies [18,19]. Upon stimulation with its endogenous specific ligand, angiotensin II (AngII), AT1R couples mainly to G_q/11_, and also possibly to G_12/13_, and engages β-arrestin with comparable efficacy for the two pathways [20,21,22]. Several peptide analogs of AngII have been developed to act as biased ligands targeting the AT1R [20,21]. Mechanistic studies of AT1R-biased agonism have been mainly focused on receptor conformation. Evidence from BRET and single molecule force spectroscopy has revealed distinct receptor conformations induced by balanced and biased ligands [23,24]. Furthermore, extant research revealed that AT1R exhibits the capacity to form homologous dimers/oligomers with distinct conformations and ligand binding sites by the in vitro assay of ensemble receptors [25,26,27,28,29], and the biological consequences of oligomerization questioned by several studies have been discussed [25,27,30,31]. Nonetheless, the association between the stoichiometry and dynamics properties of AT1R and its biased activation has not been investigated yet.

To investigate the molecular mechanism of the AT1R-biased activation process, we employed the single-molecule fluorescence imaging and tracking approaches we previously reported [32,33,34] and the cellular system stably expressing fluorescent protein-tagged AT1R we currently developed, to examine the native status of AT1R in situ after treatment by canonical ligand AngII or different biased ligands. Our findings revealed that, in resting cells, AT1R exists as monomers and spontaneously aggregated dimers and oligomers at the plasma membrane. Upon stimulations with balanced ligand AngII or biased ligand, these monomers further aggregate, resulting in slower diffusion of AT1R. Notably, a more substantial degree of oligomerization and reduced diffusion coefficient were correlated with biased activation of the β-arrestin pathway than the Gq-biased pathway. Interventions targeting downstream signaling pathways provided additional support for this conclusion. Specifically, in β-arrestin knockout cells, the heightened aggregation of AT1R in response to both AngII and biased ligands was entirely abolished, whereas the effect of G protein inhibitors was comparatively weaker. Thus, our study provides direct observations of the molecular behaviors during AT1R-biased activation in living cells at the single-molecule level. These findings indicate that AT1R’s molecular behavior reflects its biased functional states, which unveils new opportunities for the design of functionally selective drugs that can improve patient outcomes while minimizing adverse effects.

## 2. Results

### 2.1. AT1R Molecules Underwent Aggregation during AngII Activation, Which Is Inhibited by AT1R Blockers

**Figure 1:**
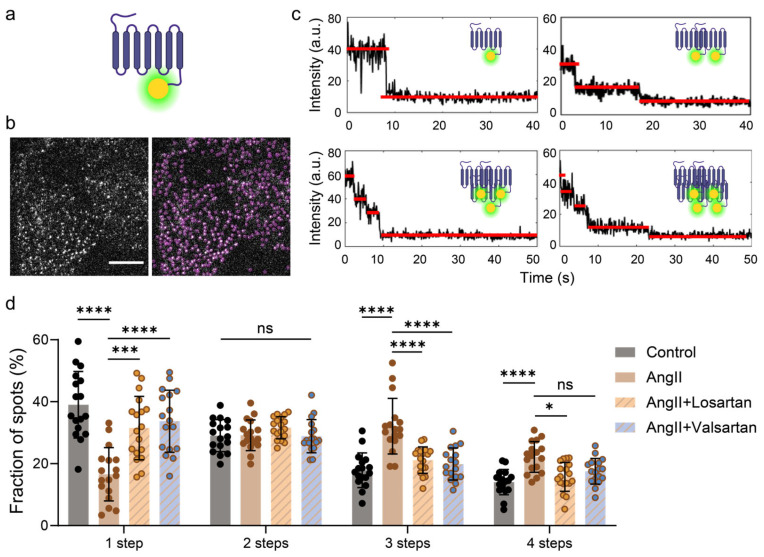
AT1R molecules underwent aggregation during AngII activation. (**a**) Schematic representation of EYFP-labeled AT1R. (**b**) A representative single-molecule fluorescence image shows the distribution of AT1R-EYFP molecules on the plasma membrane of a HeLa cell imaged by TIRFM. The image was generated by averaging the first five frames of a movie, and then the background was subtracted. The diffraction-limited spots with magenta circles (5 × 5 pixels) represented the detected individual AT1R-EYFP molecules. Scale bar, 10 μm. (**c**) Representative time courses of AT1R-EYFP emission showed multi-step photobleaching traces of AT1R. The red lines denote the automatically estimated bleaching steps. (**d**) The frequency of each step bleaching event for ATIR-EYFP in resting, ligand-stimulated, or drug-treated cells represents the fraction of AT1R-EYFP monomer, dimer, or oligomer, respectively. Data represent mean ± SD from individual AT1R molecules in approximately 15 cells each. Statistical significance was tested by two-way ANOVA with Tukey’s multiple comparisons test. **** *p* < 0.0001; *** *p* < 0.001; * *p* < 0.05; ns: no significance. The horizontal lines in the bar graph represent the groups included with no significant difference compared to control.

To better understand AT1R’s molecular behaviors, which are challenging to assess in biochemical assays due to the low expression levels of AT1R (Figure S1), we utilized single-molecule fluorescence imaging to investigate the stoichiometry of AT1R during activation by AngII in situ with living cells. Since HeLa cells endogenously express very low level of AT1R (Figure S1a), the interference from endogenous receptors is negligible. We first employed the lentiviral tool to integrate the AT1R fragment fused with EYFP into the HeLa cells’ genome, constructing a HeLa cell line stably expressing AT1R-EYFP. The cells expressing AT1R-EYFP as well-dispersed diffraction-limited fluorescent spots under TIRFM on the plasma membrane (<0.25 fluorescent spots per μm^2^) were selected for recording [35,36] (Figure 1a,b). The recorded fluorescence intensity trajectories of AT1R-EYFP over time (Figure 1c) were analyzed by a deep learning-based method, CLDNN, previously developed by us to determine stoichiometry [32]. Through our statistical analyses of the photobleaching traces, we found that in resting HeLa cells, only approximately 32.78% of AT1R molecules exhibited one-step photobleaching characteristics, indicating they were monomers. Conversely, a substantial proportion of particles displayed two, three, or even four stepwise drops, signifying dimerization, trimerization, or tetramerization at the plasma membranes (Figure 1d). These results support the view in ensemble receptors that AT1R can spontaneously form dimers or oligomers [25].

In contrast to previous reports suggesting that the aggregation state of AT1R is not affected by agonists or antagonists [25], our results indicated that the AT1R dimerization and oligomerization induced by its endogenous specific ligand AngII was very potent. For instance, exposure to a concentration of 10^−7^ M AngII reduced the proportion of monomers from the initial 39.03% to 16.57% (Figure 1d). 

Excessive AT1R activation precipitates in myocardial hypertrophy and arrhythmias [19]. Traditional AT1R blockers (ARBs) such as losartan and valsartan function by concomitantly inhibiting G-protein-mediated and β-arrestin (βarr)-mediated signaling initiated by AT1R, subsequently reducing the vasoconstrictive impact of AngII [37,38]. In our single-molecule imaging experiments, losartan and valsartan treatment restored the proportion of AT1R monomers to levels comparable to the control group (Figure 1d). Simultaneously, the number of dimers and oligomers decreased. The molecular behaviors of AT1R observed through our single molecular imaging were consistent with their biological effects, suggesting a close association between AT1R dimerization/oligomerization and ligand-induced downstream signal activation. 

Therefore, we have effectively labeled AT1R and observed different aggregation states of AT1R at the single-molecule level in situ. The monomeric AT1R underwent oligomerization after activation by the natural ligand AngII. Meanwhile, AngII-induced oligomerization is suppressed by the conventional inhibitors Losartan and Valsartan. These different states of AT1R may give rise to unique modes of action and signaling processes in response to AngII or drugs.

### 2.2. AT1R Molecules Underwent Different Aggregation States during Biased Ligand Activation

**Figure 2:**
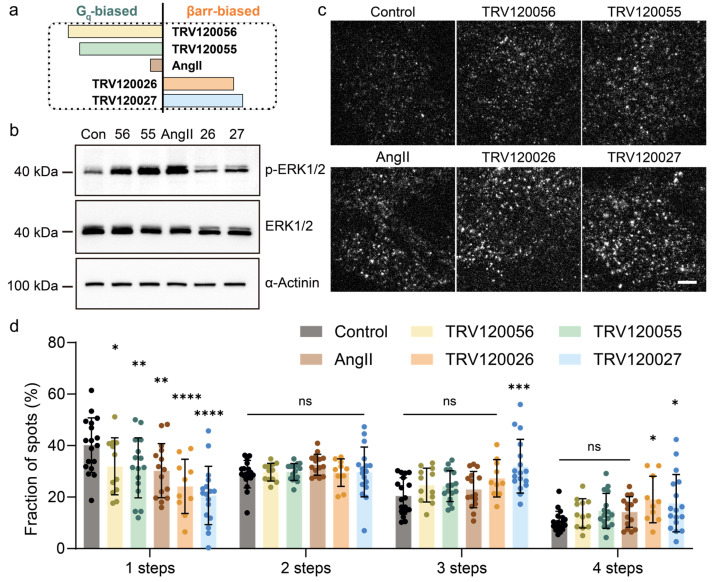
AT1R molecules underwent different aggregation states during biased ligand activation. (**a**) The schematic illustration of ligand bias. (**b**) Effect of different ligands (TRV120055, TRV120056, AngII, TRV120026, and TRV120027) on activating the ERK pathway and representative proteins of p-ERK and ERK in HeLa cells. (**c**) The representative single-molecule fluorescence images after different ligand stimulations on the HeLa cells. Scale bar, 5 μm. (**d**) The frequency of each step bleaching events for AT1R-EYFP in resting and ligand-stimulated cells. Data represent mean ± SD. Each group represents data from individual AT1R molecules in approximately 20 cells. Statistical significance was tested by two-way ANOVA test with Dunnett’s multiple comparisons test, compared with control. **** *p* < 0.0001; *** *p* < 0.001; ** *p* < 0.01; * *p* < 0.05; ns: no significance. The horizontal lines in the bar graph represent the groups included with no significant difference compared to control.

Previous investigations suggest that βarr-biased agonism may outperform ARBs, as they activate cardio-protective pathways and counteract the detrimental effects of chronic dysfunction [39,40,41,42,43]. To investigate whether the degree of different signaling pathway activation by biased ligands correlates with the AT1R molecular behaviors, we used G protein-biased agonists (TRV120055, TRV120056) that selectively trigger G protein-dependent signaling pathways and exhibited greater potency than βarr-mediated pathways. We also chose TRV120026 and TRV120027 as β-arrestin-biased agonists that elicit βarr transducer bias (Figure 2a) [20,21]. 

Extracellular signal-regulated kinase 1/2 (ERK1/2) emerges as a crucial effector in the AT1R signaling cascade. Upon ligand stimulation, at least three different downstream AT1R signaling pathways come into play, including the G-protein-coupled protein kinase C-dependent pathway, the βarr-dependent pathway, and the epidermal growth factor receptor (EGFR) transactivation pathway. These pathways collectively contribute to the phosphorylation and activation of ERK [43]. We investigated changes in cellular proteins, including ERK1/2 and p-ERK1/2, after treating HeLa cells stably expressing AT1R-EYFP with several AT1R ligands for 10 min. The phosphorylation levels of ERK1/2 proteins rose to various degrees compared to the control group, indicating that the AT1R had been successfully activated by all five ligands (Figure 2b). Notably, in contrast to the βarr-biased ligands TRV120026 and TRV120027, the balanced ligand AngII and G protein-biased ligands TRV120055 and TRV120056 led to more pronounced increases in ERK1/2 phosphorylation. These findings align with previous reports on the biased and balanced activation of AT1R [44]. G protein-biased ligands predominantly induce ERK phosphorylation through the G protein-mediated pathway, involving the activation of RAF-MEK-ERK signaling cascades. This results in a rapid and transient ERK activation. β-arrestin-biased ligands, through scaffolding for a variety of kinases, such as the mitogen-activated protein kinase (MAPK) pathway components, ultimately lead to sustained and typically more prolonged ERK activation [44]. The observed differences in increased downstream ERK phosphorylation upon AT1R activation suggest that the two types of biased ligands selectively activate specific downstream pathways, corresponding to different downstream effects.

To investigate whether the functional effects of ligand-biased activation are correlated with AT1R molecular behaviors, we employed single-molecule imaging to analyze AT1R aggregations and dynamics. AT1R following treatments was first captured in representative single-molecule images. In comparison to stimulations by TRV120055 or TRV120056, treatment with the balanced ligand AngII and the β-arrestin-biased ligands TRV120026 and TRV120027 resulted in a more pronounced aggregation of AT1R. This was evidenced by the clustering of AT1R molecules, forming more substantial and brighter puncta (Figure 2c). Quantitative photobleaching step analysis of AT1R aggregates following various ligand stimulations revealed a significant decrease in the population of AT1R monomers, accompanied by an increase in dimers and higher-order oligomers. Nevertheless, it is crucial to note that the magnitude and profile of alterations in the aggregation state of AT1R exhibited pronounced distinctions across the spectrum of ligands under investigation. βarr-biased agonists promoted AT1R to aggregate to higher-order oligomers than G protein-biased ligands. Typically, upon TRV120027 stimulation, the proportion of monomers decreased from 40.16% to 20.63%, with minimal change in the proportion of dimers (29.76%). However, there was a substantial increase in the proportions of trimers and tetramers (from 20.42% to 31.99% and 10.51% to 17.61%, respectively). In contrast, when receptors were stimulated by TRV120055, 31.36% of the AT1R entities remained as monomers, with no substantially increased dimers and oligomer fraction (29.77% dimers, 24.20% trimers, and 14.67% tetramers). This suggests that G protein-biased ligands such as TRV120055 induced weaker AT1R aggregation, and the AT1R monomers were distributed more evenly into dimers, trimers, and tetramers (Figure 2d).

**Figure 3:**
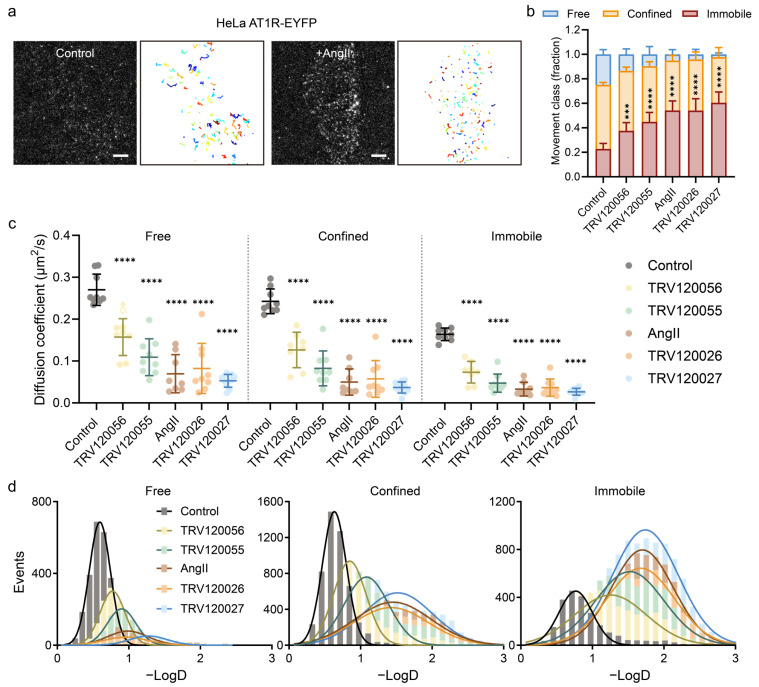
Distinct biased ligands all cause an apparent decrease in the AT1R diffusion coefficient, but the extent of their effects varies. (**a**) Typical single-molecule imaging and tracking of AT1R-EYFP molecules at the plasma membrane of living HeLa cells. Scale bar, 5 μm. (**b**) Stacked Histogram shows fractions of AT1R tracks classified as free, confined, or immobile under diverse biased ligand stimulations. (**c**) The diffusion coefficient values for free, confined, and immobile diffusion states of AT1R-EYFP molecules under different biased ligand stimulations. Data represent mean ± SD. Approximately 10 cells each in control, different ligand-stimulated groups were selected for analysis (**b**,**c**). Statistical significance was tested by one-way ANOVA with Dunn’s multiple comparisons test, compared with control (**b**,**c**). **** *p* < 0.0001; *** *p* < 0.001. (**d**) Distributions of the diffusion coefficient of membrane-docked AT1R-EYFP molecules in each mode of motion after diverse biased ligand stimulations.

We further tracked the lateral diffusion of individual AT1R molecules on the living cell membrane (Figure 3a). Automatic single-molecule detection and tracking revealed that the majority of AT1R molecules exhibited free or confined diffusion at the plasma membranes of resting HeLa cells. However, upon stimulation with all ligands, there was a partial transition of free and confined diffusing AT1Rs to an immobile state (Figure 3b). Notably, the βarr-biased ligands TRV120026 and TRV120027 induced a more substantial increase in the proportion of immobile AT1R compared to the balanced ligand AngII and the G protein-biased ligands TRV120055 and TRV120056. Quantitative analysis of diffusion coefficients for thousands of individually tracked AT1R-EYFP molecules showed that stimulation with all five ligands led to a shift in the distribution of diffusion coefficients (D) of AT1R molecules toward lower values across all three diffusion modes (Figure 3c). Importantly, the βarr-biased ligands induced a more pronounced slowing of AT1R diffusion compared to the G protein-biased ligands in all three diffusion modes. For instance, the diffusion coefficient of free AT1R decreased from 0.2703 ± 0.0373 μm^2^/s in resting cells to 0.1092 ± 0.0439 μm^2^/s in TRV120055-stimulated cells and 0.0528 ± 0.0154 μm^2^/s in TRV120027-stimulated cells (Figure 3d).

Considering that the diffusive behaviors of membrane-bound proteins are influenced by the size of their membrane-bound regions [45], the observed slower diffusion coefficient of AT1R implies that they underwent higher-order oligomerization induced by β-arrestin-biased ligands. These tracking results were in accordance with our findings from photobleaching analyses. Consequently, our data indicated that both G-protein-biased and βarr-biased ligand actions are closely linked to ligand-induced AT1R aggregation. Moreover, a higher degree of AT1R aggregation appears to be particularly associated with βarr-dependent signaling, suggesting that the particular aggregation and dynamic of AT1R molecules reflect their biased functional activation states.

### 2.3. Gq Signaling Had Negligible Effect on AT1R Oligomerization, While Slightly Immobilized Molecule Diffusion

**Figure 4:**
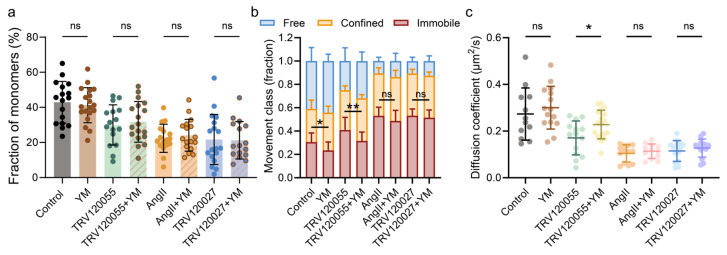
G protein signaling had negligible effect on AT1R oligomerization, while slightly accelerated molecule kinetics. (**a**) Frequency of one-step bleaching events for AT1R-EYFP in resting, ligand-stimulated, and inhibitor-treated cells. Data represent mean ± SD from individual AT1R molecules in approximately 15 cells each. (**b**) Stacked Histogram shows fractions of AT1R tracks classified as free, confined, or immobile with or without ligands or inhibitors treatment. (**c**) The diffusion coefficient values for AT1R-EYFP molecules from HeLa cells under different conditions as set in (**b**). Data represent mean ± SD. Approximately 10 cells each in control, ligand- or other inhibitor-treated groups were selected for analysis (**b**,**c**). Statistical significance was tested by unpaired *t*-test (**a**–**c**). ** *p* < 0.01; * *p* < 0.05; ns: no significance.

Next, we aimed to confirm the relationship between different biased pathways with AT1R molecular behavior, focusing on the downstream effector proteins. First, we investigated the effect of the G protein-biased signaling for the receptor dimers/oligomers observed above. Since AT1R exerts G protein signaling notably through Gq-mediated activation, the YM-254890 (YM) was introduced to inhibit Gq protein function (Figure S2). We found that YM had little effect on the assembly of AT1R into dimers or oligomers following stimulations with the balanced ligand AngII, the G protein-biased ligand TRV120055, and the βarr-biased ligand TRV120027 (Figure 4a). 

To further examine the impact of G protein binding on molecular behaviors of AT1R upon ligand stimulation, we investigated the diffusive dynamics of AT1R. Our results demonstrated that changes in diffusive modes and coefficients induced by the activation of G protein-biased ligand TRV120055, but not AngII and βarr-biased ligand TRV120027, were slightly attenuated by pretreatment with YM. More specifically, the introduction of YM exerts a slight inhibitory effect on the TRV120055-induced transition of AT1R, steering it away from a relatively immobile diffusion mode, toward free and confined diffusion modes, while the influence of YM appears to be negligible when juxtaposed with its impact on AT1R molecules in response to AngII and TRV120027 stimulations (Figure 4b). Simultaneously, YM treatment emerges as a partial impediment to the attenuation of diffusion dynamics in AT1R molecules induced by TRV120055. In a notable contrast, this treatment exhibits no discernible influence on the diffusion dynamics of AT1R molecules in the context of AngII and TRV120027 stimulations (Figure 4c). These results suggested that Gq-mediated signaling transduction had no effect on AT1R stoichiometry, but altered their diffusion dynamics, producing different effects on AT1R dynamics according to stimulations with a distinct biased ligand, which likely correlated with the altered transmembrane conformation of AT1R during G protein binding.

### 2.4. β-Arrestin Recruitment Is a Critical Step in Ligand-Induced AT1R Aggregation

**Figure 5:**
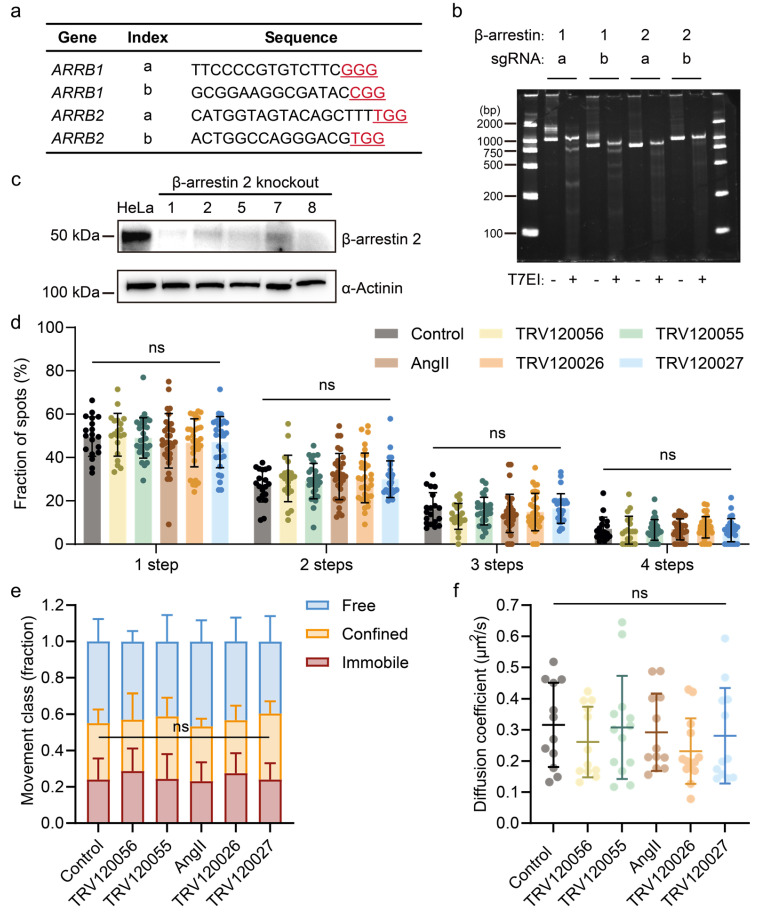
The binding of β-arrestin was a critical step in AT1R aggregation upon distinct biased ligand stimulations. (**a**) The sequence of the designed *sg*RNA. (**b**) T7EI assay for validating *sg*RNA activities targeting βarr 1/2. Index a and b indicate *sg*RNA targeting different sites. (**c**) Western blot showing the cellular expression levels of βarr2 in knock-out cell lines. (**d**) Fraction of photobleaching step numbers for all AT1R-EYFP molecules analyzed in βarr1/2 knock-out cell upon distinct biased ligand stimulations. Each group represents data from individual AT1R molecules in approximately 20 cells. (**e**) The fractions of AT1R tracks classified as free, confined, or immobile and (**f**) the diffusion coefficient values for AT1R-EYFP molecules with or without ligands treatment in approximately 10 βarr1/2 knock-out cells each. Data represent mean ± SD (**d**–**f**). Statistical significance was tested by two-way ANOVA with Dunnett’s multiple comparisons test (**d**) and one-way ANOVA with Dunnett’s multiple comparisons test (**e**,**f**). ns: no significance. The horizontal lines in the bar graph represent the groups included with no significant difference compared to control.

We further investigated the role of βarr in AT1R-biased activation using βarr1/2 knockout HeLa cells through CRISPR/Cas9 genome-editing, in comparison to the parental HeLa cells. HeLa cells originally do not express endogenous βarr1 (Figure S3). To ensure ontogenetic cleanliness, we still designed two stripes of *sg*RNA for each protein (βarr1 and βarr2) to achieve site-specific genome editing (Figure 5a). The genome-editing efficiency by CRISPR/Cas9 targeting in HeLa cells was examined with genomic DNA by T7 Endonuclease I (T7EI) analysis. Agarose gel electrophoresis performed on the T7EI digested DNA showed bands of cut-down DNA in four groups of DNA amplifying the four *sg*RNA targeting sites respectively, confirming successful *sg*RNA activities at the βarr1 and βarr2 loci with Cas9 (Figure 5b). The genome-edited cells were subsequently sorted into single-cell lines using flow cytometry to obtain cell lines with a uniform genotype. To verify βarr2 expression levels in these cell lines, we performed Western blot analysis and identified the cell lines that exhibited no βarr2 expression for subsequent experiments (Figure 5c).

Single-molecule imaging of individual AT1R-EYFP molecules in these βarr 1/2 knockout HeLa cells displayed no significant differences before and after stimulation with any of the five ligands (Figure S4). Consistent with the imaging results, photobleaching step analysis demonstrated that βarr 1/2 knockout completely blocked the ligand-induced oligomerization of AT1R on the cell membrane, irrespective of ligand bias (Figure 5d). These findings suggest that βarr recruitment is not a step that engages transduction signals after biased ligand-stimulated AT1R oligomerization. Instead, it appears to be a crucial effector responsible for mediating receptor aggregation in response to ligand stimulation. The influence of βarr is not limited solely to βarr-biased ligand stimulation but is similarly evident in G protein-biased ligand stimulation. This is likely due to the pivotal role of βarr in regulating receptor internalization [22]. These results are consistent with previous experimental observations that the stabilization of AT1R homodimer through cross-linking increased inositol phosphate (IP) levels (enhanced signaling) and the internalization of cross-linked AT1R dimers [26].

Furthermore, both G protein-biased and βarr-biased ligands induced diffusion slowdown, and the movement mode transition of AT1R was totally abrogated in βarr 1/2 knockout HeLa cells (Figure 5e,f). These results suggested that the diffusive dynamic change of AT1R molecules in ligand-induced agonism (regardless of signaling orientation) was primarily regulated by βarr. 

## 3. Discussion

The bidirectional interplay between G proteins and β-arrestin has been a focal point of extensive investigation in prior research. Since Hern et al. and Kasai et al. employed single-particle tracking (SPT) analysis using fluorescent ligands to reveal the transient feature of GPCR dimerization, the stage for a more detailed exploration of GPCR behavior at the single-molecule level was set [46,47]. In recent years, emerging evidence suggests that the aggregation state of GPCRs occupies a pivotal position in modulating biased signaling [13]. 

Nevertheless, it is imperative to acknowledge that there is still controversy and knowledge gaps regarding these mechanisms, as different GPCRs, even belonging to the same class, exhibited opposite aggregation behaviors in response to different biased signaling [13,15]. In this work, we employed single-molecule imaging and tracking techniques to reveal the molecular behaviors underlying biased signaling in GPCRs, with a focus on AT1R. It is now evident that AT1R exists in multiple aggregation states, including major monomers, dimers, and minor oligomers, and that ligand-induced activation has a significant impact on its aggregation profile. This observation is particularly important as it challenges the conventional view that GPCRs exist primarily as monomers and compellingly demonstrates the intricately interconnected nature of both G-protein-biased and β-arrestin-biased ligand actions, revealing their intimate association with the inducement of AT1R aggregation. 

Notably, we found that AT1R exhibited a higher degree of aggregation in response to β-arrestin-biased ligands compared to G protein pathway activation, suggesting that AT1R aggregation, a phenomenon previously reported for other GPCRs, may be associated with the selectivity and specificity of downstream signaling pathways. These results also confirmed that β-arrestin signaling, but not G protein signaling, plays a significant and indispensable role in ligand-induced AT1R aggregation, suggesting that β-arrestin recruitment is not merely a downstream consequence of biased ligand-stimulated AT1R oligomerization but a fundamental effector of receptor aggregation. This evidence of the link between AT1R’s molecular oligomerization and its biased activation offers new information for the rational design of biased ligands targeting AT1R. By understanding how specific ligands influence AT1R aggregation and activation, drug developers may be able to fine-tune ligand properties to achieve desired therapeutic outcomes with minimal side effects.

Another interesting observation of our study is that ligand stimulations, regardless of the bias, commonly decreased the diffusion coefficient of AT1R, but to varying degrees, and that the immobilization of AT1R upon activation is primarily related to β-arrestin. Traditionally, the change in receptor mobility is in intimate contact with the change in receptor aggregation, which causes the change in contact area between the protein and the membrane. In this study, our observed changes in AT1R diffusion dynamics induced by biased ligands were highly aligned with the aggregation state, and the higher the order of oligomerization, the slower the diffusion. Emerging evidence suggests that the local membrane environment influenced by cytoskeleton, lipid rafts, or transducers is significantly impacted. The dual-color SPT analysis uncovered membrane domains where adrenergic receptors and G proteins co-accumulate, forming signaling hot spots [48]. Regarding AT1R, Kawakami et al. conducted dual-color single-molecule imaging, revealing the differential regulation of AT1R diffusion by different agonists, dependent on the selectivity of transducer (GRK) subtypes and β-arrestin binding modes [49]. Further investigations into combination with multicolor labeling, including membrane lipids and intra-membrane effectors, are needed to investigate whether the diffusion dynamic of AT1R affected by biased ligands is a combined result of a multifactorial network of interactions.

AT1R is a significant therapeutic target in cardiovascular pharmacotherapies. It is conceivable that the dynamic transition between AT1R monomers, dimers, and oligomers of AT1R also regulates the biased biological activity of the AT1R signaling pathway and plays an important regulatory role in cardiovascular disease. Understanding how ligands influence AT1R aggregation and downstream signaling contributes to elucidating the pathogenesis of cardiovascular diseases.

While our research has offered new insights into the differences in AT1R aggregation and diffusion in response to differential biased ligands, it is crucial to recognize that our study has not clearly provided a comprehensive understanding of the downstream signaling pathways and physiological consequences associated with these molecular differences. It is required to explore the potential correspondence of the AT1R aggregation state in the precise control of orienting one signaling over another. To address this gap, future research could involve the development of AT1R mutants designed to form stabilizing dimers or maintain absolute monomers. This approach would allow for a more targeted investigation into the specific impact of AT1R oligomerization on biased signaling, rather than applying the genome-editing tool to knock out β-arrestin, which introduces the limitation of altering the entire signaling pathway. Furthermore, further studies to distinguish the β-arrestin-regulated AT1R aggregation from β-arrestin-mediated AT1R endocytosis remain to be conducted to understand the role of β-arrestin in biased signaling. 

## 4. Materials and Methods

Cell Culture and Treatment. HeLa cells were purchased from the Cell Resource Center of Peking Union Medical College and were cultured in Dulbecco’s modified Eagle’s medium (DMEM, Gibco, Waltham, MA, USA) supplemented with 10% fetal bovine serum (FBS, Gibco) and penicillin/streptomycin (100 IU/mL, Gibco) at 37 °C in a 5% CO_2_ atmosphere.

Reagents. Angiotensin II, TRV120056, TRV120055, TRV120026, and TRV120027 were purchased from Sigma-Aldrich (Saint Louis, MI, USA) and GenScript (Piscataway, NJ, USA). YM-254890 was purchased from Macklin (Shanghai, China). Lipofectamine 3000 was purchased from Thermo Fisher, Waltham, MA, USA. Antibodies were listed as follows: anti-phospho-ERK antibody (4370, Cell Signaling Technology, Boston, MA, USA), anti-ERK antibody (4695, Cell Signaling Technology), anti-β-arrestin 1 antibody (30036, Cell Signaling Technology), anti-β-arrestin 2 antibody (3857, Cell Signaling Technology), and anti-α-actinin antibody (69758, Cell Signaling Technology).

Construction of Cell Lines Stably Expressing AT1R-EYFP. The cDNA encoding the AT1R gene was cloned into the pEYFP-N1 vector using the pEASY-Uni Seamless Cloning and Assembly Kit (Full Vision, Beijing, China). Amplicons of the AT1R-EYFP fragment were then cloned into the pLVX-Puro vector. 

The lentivirus package and stable cell lines generation proceeds as follows: The HEK293T cells were plated in complete culture media in one 60 mm dish. When the cells had reached 90% confluency, they were transfected with 3 plasmids, including pLVX-Puro, pMD2.G, and psPAX2, using the X-tremeGENE HP DNA Transfection Reagent. After 6 h of transfection, the supernatant was changed with a culture medium containing 30% FBS. Virus-containing supernatants were collected every 48 h and 72 h, pooled together, and filtered using a filter membrane with a pore size of 0.45 mm to remove the debris from HEK293T cells. Hela cells with 70% confluency were incubated in a 1:1 ratio of viral supernatant to complete medium, and polybrene was added to reach a final concentration of 10 ug/mL for 12 h.

Construction of β-Arrestin Knockout Cell Lines. The lentiCRISPR V2 plasmid (#52961, Addgene, Watertown, MA, USA) underwent precise enzymatic digestion utilizing BsmBI, followed by the meticulous integration of designed and annealed oligonucleotides, seamlessly embedded within the single guide RNA scaffold. The resultant construct was subsequently deployed to specifically target and highly define genomic loci of interest by being packaged as lentiviruses for further use in infecting HeLa cells. After infection, puromycin selection was conducted, and surviving cells were subjected to FACS sorting.

Ligand Stimulation. For the biased ligand stimulation assay, Cells grown in a 6-well plate were serum-starved overnight and allowed to reach 70–90% confluence prior to stimulations. AngII and other biased ligands (Table 1) were added to the cell culture medium to achieve a final concentration of 100 nM for 10 min in a 37 °C incubator. For the AT1R blockers (ARBs) group, Losartan or Valsartan were pre-added to the medium to achieve a final concentration of 40 µM, incubated for 20 min, and AngII was added to a final concentration of 100 nM for 10 min. For the AT1R inhibitor group, cells were pretreated by 10 μM YM-254890 for 30 min and AngII or other biased ligands were added to a final concentration of 100 nM for 10 min.

Single-Molecule Fluorescence Imaging. To detect individual AT1R on the cell membrane, the HeLa cells that stably expressed AT1R-EYFP were seeded in 35 mm glass-bottomed culture dishes coated with poly-D-lysine (PDL). Then, HeLa monolayers (75% confluent) previously serum-starved overnight were stimulated for 10 min and directly observed for tracking. For data acquisition in the photobleaching steps, cells were washed twice with DPBS and fixed with a solution of 3% (wt/vol) paraformaldehyde and 0.1% (wt/vol) glutaraldehyde in DPBS for 20 min, followed by three DPBS washes before imaging.

Single-molecule fluorescence imaging was performed using homemade total internal reflection fluorescence microscopy (TIRFM) according to previous reports [50]. The TIRFM setup was mounted on an inverted Olympus IX71 microscope and included a total internal reflective fluorescence illuminator, a 100X/1.45NA plan-apochromatic objective lens (Olympus), and a single-photon detector 14-bit EMCCD (Andor iXon DU-897 BV). An argon ion laser (Melles Griot, Carlsbad, CA, USA) at 488 nm was used to excite EYFP. The excitation power used in experiments was 1 mW (measured at the objective output), corresponding to 17 W/cm^2^. The fluorescent signal was gathered by the same objective. Additionally, the Semrock notch filter (NF01-488) was used to block dispersed laser light. The EMCCD’s electron-multiplying gain was set to 300. Images were acquired using IQ software 3 (Andor, BT) at a frame rate of 100 ms/frame, each consisting of 400 consecutive frames.

Single-Molecule Fluorescence Bleaching Trajectory Extraction. The method for extracting single-molecule fluorescence bleaching trajectories was adapted from the processing techniques described in a previously published paper [15]. Initially, background fluorescence was subtracted using the Rolling Ball method (12/15 pixels) in Image J software v1.54f (http://rsb.info.nih.gov/ij/, accessed on 27 November 2023), followed by averaging the first five frames. Based on the point spread function (PSF) of the imaging system, the approximate size of a single-molecule fluorescent spot was approximately 5 × 5 pixels (with each pixel representing 160 nm). Subsequently, a 2D Gaussian fitting was applied to each frame, and single-molecule spots that exhibited a Gaussian distribution were recorded according to previous reports [51]. To be considered as candidate points, a spot needed to have a signal-to-noise ratio exceeding a predefined threshold and should persist for at least 5 frames. However, points that were too close to each other (less than 4 pixels apart) or exhibited elliptical shapes (eccentricity > 1.2) were excluded. Roundness was defined as follows [52]:

*eccentricity* = max (*wx*, *wy*)/min (*wx*, *wy*)

For the final single-molecule points that met these criteria, their fluorescence intensities were recorded frame by frame, resulting in bleaching curves representing fluorescence intensity over time, which were used for photobleaching event counting analysis. However, some trajectories exhibited a sloping behavior with no distinct stepwise decrease in fluorescence intensity. To address this, a polynomial function was employed to fit these trajectories, and those with an R-squared value > 0.83, indicating a close approximation to a slope, were considered challenging to analyze and thus excluded.

The photobleaching event counting was performed by convolutional and long-short-term memory deep learning neural network (CLDNN) [32]. 

Single-Molecule Fluorescence Tracking and Diffusion Analysis. Individual AT1R-EYFP tracking images were acquired at a frame rate of 10 Hz, forming a time-lapse series consisting of 300 frames. A powerful tracking tool (trackmate) was used to detect and track individual receptor molecules [53]. The parameters with non-default values used a diameter of 2.5 pixels; a threshold of 180; a max distance of 7 pixels; a gap-closing max distance of 7 pixels; and a gap-closing max frame distance of 2 pixels. For the purpose of calculating the diffusion coefficient, the following formulas were used to create the two-dimensional mean square displacement (MSD) for each time interval tn of each trajectory [54]:

(1) $$\mathrm{MSD}(n\delta t)=\frac{1}{N-1-n}\overset{N-1-n}{\underset{i=1}{\sum }}\left\{{\left[x(i\delta t+n\delta t)-x(i\delta t)\right]}^{2}+{\left[y(i\delta t+n\delta t)-y(i\delta t)\right]}^{2}\right\}$$

where Δt_n_ is the amount of time it takes for a single AT1R-EYFP molecule to migrate from position *x* (*iδt*), *y* (*iδt*), to position *x* (*iδt + nδt*), *y* (*iδt + nδt*), with *δt* = 100 ms. Integers *n* and *I* determine the time increment and *n*. The overall number of image frames is *N*. The diffusion coefficient (D) of each molecule was calculated using the least-squares fitting method using the slope of the first four points in the MSD-Δt plot and the equation MSD(Δt) = 4DΔt. The upper limit of the lag time n used in the calculation of MSD is 5. The classification of the movements of individual tracks was accomplished using DC-MSS [55].

Western Blotting. HeLa cells were lysed in RIPA buffer containing protease and phosphatase inhibitors (Thermo Fisher Scientific) for 10 min on ice, spun in a pre-cooled centrifuge (4 °C) for 5 min (14,000 rpm), and quantified using the BCA Protein Assay Kit (Bytotime Biotechnology, Shanghai, China). The collected proteins were boiled in 5× sample buffer for 5 min, separated by SDS-PAGE, and then transferred onto NC membranes (Millipore, Burlington, MA, USA). After blocking with 5% skim milk (BD, Franklin Lakes, NJ, USA) for 1 h, the membranes were incubated overnight with primary antibodies at 4 °C, followed by incubation with secondary antibodies at room temperature for 1 h. Finally, the protein bands were visualized using a super enhanced chemiluminescence detection reagent (Applygen, Beijing, China).

T7 Endonuclease I (T7EI) assay. Genomic DNA was isolated from HeLa cells treated with *sg*RNA-Cas9. Samples collected were used as the template for PCR using self-designed primers (Figure S5). Amplicons of the β-arrestin genomic fragment obtained from those samples were denatured, renatured, and treated with T7EI (New England Biolabs, Ipswich, MA, USA) according to the manufacturer’s protocol. Results are analyzed by visualizing cleavage products and full-length amplicons by gel electrophoresis. 

Statistical Analysis. GraphPad Prism software (9.0.0) was used to assess statistical significance. Unpaired for comparisons between two groups, the Student’s *t*-test was used, and for comparisons between more than two groups, the one-way ANOVA was used. For comparisons between more than two multi-factor groups, the two-way ANOVA test was applied. All statistical analyses were conducted on a two-sided basis, and statistical significance was determined at the *p* < 0.05 level.

## Figures and Tables

**Table 1:** AT1R ligands and sequence information.

Ligand	Peptide Sequence
TRV120056	Asp-Arg-Gly-Val-Tyr-Ile-His-Pro-Phe
TRV120055	Gly-Val-Tyr-Ile-His-Pro-Phe
AngII	Asp-Arg-Val-Tyr-Ile-His-Pro-Phe
TRV120026	Sar-Arg-Val-Tyr-Tyr-His-Pro-NH2
TRV120027	Sar-Arg-Val-Tyr-Ile-His-Pro-D-Ala

## Data Availability

The datasets used and/or analyzed during the current study are included in this article and available from the corresponding author upon reasonable request.

## Supplementary Materials

The following supporting information can be downloaded at: https://www.mdpi.com/article/10.3390/ijms25010374/s1.

## References

[1] Hauser A.S., Attwood M.M., Rask-Andersen M., Schiöth H.B., Gloriam D.E. (2017). Trends in GPCR drug discovery: New agents, targets and indications. Nat. Rev. Drug Discov..

[2] Hauser A.S., Chavali S., Masuho I., Jahn L.J., Martemyanov K.A., Gloriam D.E., Babu M.M. (2018). Pharmacogenomics of GPCR Drug Targets. Cell.

[3] Wootten D., Christopoulos A., Marti-Solano M., Babu M.M., Sexton P.M. (2018). Mechanisms of signalling and biased agonism in G protein-coupled receptors. Nat. Rev. Mol. Cell Biol..

[4] Ahn S., Shenoy S.K., Luttrell L.M., Lefkowitz R.J. (2020). SnapShot: β-Arrestin Functions. Cell.

[5] Slosky L.M., Caron M.G., Barak L.S. (2021). Biased Allosteric Modulators: New Frontiers in GPCR Drug Discovery. Trends Pharmacol. Sci..

[6] Yang D., Zhou Q., Labroska V., Qin S., Darbalaei S., Wu Y., Yuliantie E., Xie L., Tao H., Cheng J. (2021). G protein-coupled receptors: Structure- and function-based drug discovery. Signal Transduct. Target. Ther..

[7] Seyedabadi M., Ghahremani M.H., Albert P.R. (2019). Biased signaling of G protein coupled receptors (GPCRs): Molecular determinants of GPCR/transducer selectivity and therapeutic potential. Pharmacol. Ther..

[8] Klein Herenbrink C., Sykes D.A., Donthamsetti P., Canals M., Coudrat T., Shonberg J., Scammells P.J., Capuano B., Sexton P.M., Charlton S.J. (2016). The role of kinetic context in apparent biased agonism at GPCRs. Nat. Commun..

[9] Wingler L.M., Lefkowitz R.J. (2020). Conformational Basis of G Protein-Coupled Receptor Signaling Versatility. Trends Cell Biol..

[10] Wacker D., Stevens R.C., Roth B.L. (2017). How Ligands Illuminate GPCR Molecular Pharmacology. Cell.

[11] Ge B., Lao J., Li J., Chen Y., Song Y., Huang F. (2017). Single-molecule imaging reveals dimerization/oligomerization of CXCR4 on plasma membrane closely related to its function. Sci. Rep..

[12] Nishiguchi T., Yoshimura H., Kasai R.S., Fujiwara T.K., Ozawa T. (2020). Synergetic Roles of Formyl Peptide Receptor 1 Oligomerization in Ligand-Induced Signal Transduction. ACS Chem. Biol..

[13] Liu J., Tang H., Xu C., Zhou S., Zhu X., Li Y., Prézeau L., Xu T., Pin J.-P., Rondard P. (2022). Biased signaling due to oligomerization of the G protein-coupled platelet-activating factor receptor. Nat. Commun..

[14] Calebiro D., Rieken F., Wagner J., Sungkaworn T., Zabel U., Borzi A., Cocucci E., Zürn A., Lohse M.J. (2013). Single-molecule analysis of fluorescently labeled G-protein–coupled receptors reveals complexes with distinct dynamics and organization. Proc. Natl. Acad. Sci. USA.

[15] Sun Y., Li N., Zhang M., Zhou W., Yuan J., Zhao R., Wu J., Li Z., Zhang Y., Fang X. (2016). Single-molecule imaging reveals the stoichiometry change of β2-adrenergic receptors by a pharmacological biased ligand. Chem. Commun..

[16] Möller J., Isbilir A., Sungkaworn T., Osberg B., Karathanasis C., Sunkara V., Grushevskyi E.O., Bock A., Annibale P., Heilemann M. (2020). Single-molecule analysis reveals agonist-specific dimer formation of µ-opioid receptors. Nat. Chem. Biol..

[17] Zhang H., Unal H., Gati C., Han Gye W., Liu W., Zatsepin Nadia A., James D., Wang D., Nelson G., Weierstall U. (2015). Structure of the Angiotensin Receptor Revealed by Serial Femtosecond Crystallography. Cell.

[18] de Gasparo M., Catt K.J., Inagami T., Wright J.W., Unger T. (2000). International union of pharmacology. XXIII. The angiotensin II receptors. Pharmacol. Rev..

[19] Kawai T., Forrester S.J., O’Brien S., Baggett A., Rizzo V., Eguchi S. (2017). AT1 receptor signaling pathways in the cardiovascular system. Pharmacol. Res..

[20] Strachan R.T., Sun J.P., Rominger D.H., Violin J.D., Ahn S., Rojas Bie Thomsen A., Zhu X., Kleist A., Costa T., Lefkowitz R.J. (2014). Divergent transducer-specific molecular efficacies generate biased agonism at a G protein-coupled receptor (GPCR). J. Biol. Chem..

[21] Rajagopal S., Ahn S., Rominger D.H., Gowen-MacDonald W., Lam C.M., Dewire S.M., Violin J.D., Lefkowitz R.J. (2011). Quantifying ligand bias at seven-transmembrane receptors. Mol. Pharmacol..

[22] Forrester S.J., Booz G.W., Sigmund C.D., Coffman T.M., Kawai T., Rizzo V., Scalia R., Eguchi S. (2018). Angiotensin II Signal Transduction: An Update on Mechanisms of Physiology and Pathophysiology. Physiol. Rev..

[23] Devost D., Sleno R., Pétrin D., Zhang A., Shinjo Y., Okde R., Aoki J., Inoue A., Hébert T.E. (2017). Conformational Profiling of the AT1 Angiotensin II Receptor Reflects Biased Agonism, G Protein Coupling, and Cellular Context. J. Biol. Chem..

[24] Li W., Xu J., Kou X., Zhao R., Zhou W., Fang X. (2018). Single-molecule force spectroscopy study of interactions between angiotensin II type 1 receptor and different biased ligands in living cells. Anal. Bioanal. Chem..

[25] Hansen J.L., Theilade J., Haunsø S., Sheikh S.P. (2004). Oligomerization of wild type and nonfunctional mutant angiotensin II type I receptors inhibits galphaq protein signaling but not ERK activation. J. Biol. Chem..

[26] AbdAlla S., Lother H., Langer A., el Faramawy Y., Quitterer U. (2004). Factor XIIIA transglutaminase crosslinks AT1 receptor dimers of monocytes at the onset of atherosclerosis. Cell.

[27] Szalai B., Barkai L., Turu G., Szidonya L., Várnai P., Hunyady L. (2012). Allosteric interactions within the AT_1_ angiotensin receptor homodimer: Role of the conserved DRY motif. Biochem. Pharmacol..

[28] Young B.M., Nguyen E., Chedrawe M.A.J., Rainey J.K., Dupré D.J. (2017). Differential Contribution of Transmembrane Domains IV, V, VI, and VII to Human Angiotensin II Type 1 Receptor Homomer Formation. J. Biol. Chem..

[29] Porrello E.R., Pfleger K.D.G., Seeber R.M., Qian H., Oro C., Abogadie F., Delbridge L.M.D., Thomas W.G. (2011). Heteromerization of angiotensin receptors changes trafficking and arrestin recruitment profiles. Cell. Signal..

[30] Guo A.-Y., Zhang Y.-M., Wang L., Bai D., Xu Y.-P., Wu W.-Q. (2021). Single-Molecule Imaging in Living Plant Cells: A Methodological Review. Int. J. Mol. Sci..

[31] Wang X., Song K., Li Y., Tang L., Deng X. (2019). Single-Molecule Imaging and Computational Microscopy Approaches Clarify the Mechanism of the Dimerization and Membrane Interactions of Green Fluorescent Protein. Int. J. Mol. Sci..

[32] Xu J.C., Qin G.G., Luo F., Wang L.N., Zhao R., Li N., Yuan J.H., Fang X.H. (2019). Automated Stoichiometry Analysis of Single-Molecule Fluorescence Imaging Traces via Deep Learning. J. Am. Chem. Soc..

[33] Zhao R., Yuan J., Li N., Sun Y., Xia T., Fang X. (2019). Analysis of the Diffusivity Change from Single-Molecule Trajectories on Living Cells. Anal. Chem..

[34] Xia T., Li N., Fang X. (2013). Single-molecule fluorescence imaging in living cells. Annu. Rev. Phys. Chem..

[35] Zhang M., Chang H., Zhang Y., Yu J., Wu L., Ji W., Chen J., Liu B., Lu J., Liu Y. (2012). Rational design of true monomeric and bright photoactivatable fluorescent proteins. Nat. Methods.

[36] Lu Y., Huang X., Wang S., Li B., Liu B. (2023). Nanoconfinement-Enhanced Electrochemiluminescence for in Situ Imaging of Single Biomolecules. ACS Nano.

[37] Timmermans P.B., Wong P.C., Chiu A.T., Herblin W.F., Benfield P., Carini D.J., Lee R.J., Wexler R.R., Saye J.A., Smith R.D. (1993). Angiotensin II receptors and angiotensin II receptor antagonists. Pharmacol. Rev..

[38] Manabe I., Shindo T., Nagai R. (2002). Gene expression in fibroblasts and fibrosis: Involvement in cardiac hypertrophy. Circ. Res..

[39] DeWire S.M., Violin J.D. (2011). Biased ligands for better cardiovascular drugs: Dissecting G-protein-coupled receptor pharmacology. Circ. Res..

[40] Revankar C.M., Vines C.M., Cimino D.F., Prossnitz E.R. (2004). Arrestins block G protein-coupled receptor-mediated apoptosis. J. Biol. Chem..

[41] Whalen E.J., Rajagopal S., Lefkowitz R.J. (2011). Therapeutic potential of β-arrestin- and G protein-biased agonists. Trends Mol. Med..

[42] Boerrigter G., Lark M.W., Whalen E.J., Soergel D.G., Violin J.D., Burnett J.C. (2011). Cardiorenal actions of TRV120027, a novel ß-arrestin-biased ligand at the angiotensin II type I receptor, in healthy and heart failure canines: A novel therapeutic strategy for acute heart failure. Circ Heart Fail..

[43] Violin J.D., DeWire S.M., Yamashita D., Rominger D.H., Nguyen L., Schiller K., Whalen E.J., Gowen M., Lark M.W. (2010). Selectively engaging β-arrestins at the angiotensin II type 1 receptor reduces blood pressure and increases cardiac performance. J. Pharmacol. Exp. Ther..

[44] Kim J., Ahn S., Rajagopal K., Lefkowitz R.J. (2009). Independent β-Arrestin2 and Gq/Protein Kinase Cζ Pathways for ERK Stimulated by Angiotensin Type 1A Receptors in Vascular Smooth Muscle Cells Converge on Transactivation of the Epidermal Growth Factor Receptor. J. Biol. Chem..

[45] Pan Q., Sun D., Xue J., Hao J., Zhao H., Lin X., Yu L., He Y. (2021). Real-Time Study of Protein Phase Separation with Spatiotemporal Analysis of Single-Nanoparticle Trajectories. ACS Nano.

[46] Hern J.A., Baig A.H., Mashanov G.I., Birdsall B., Corrie JE T., Lazareno S., Molloy J.E., Birdsall N.J.M. (2010). Formation and dissociation of M-1 muscarinic receptor dimers seen by total internal reflection fluorescence imaging of single molecules. Proc. Natl. Acad. Sci. USA.

[47] Kasai R.S., Suzuki KG N., Prossnitz E.R., Koyama-Honda I., Nakada C., Fujiwara T.K., Kusumi A. (2011). Full characterization of GPCR monomer-dimer dynamic equilibrium by single molecule imaging. J. Cell Biol..

[48] Sungkaworn T., Jobin M.-L., Burnecki K., Weron A., Lohse M.J., Calebiro D. (2017). Single-molecule imaging reveals receptor–G protein interactions at cell surface hot spots. Nature.

[49] Kawakami K., Yanagawa M., Hiratsuka S., Yoshida M., Ono Y., Hiroshima M., Ueda M., Aoki J., Sako Y., Inoue A. (2022). Heterotrimeric Gq proteins act as a switch for GRK5/6 selectivity underlying β-arrestin transducer bias. Nat. Commun..

[50] Zhang W., Jiang Y.X., Wang Q., Ma X.Y., Xiao Z.Y., Zuo W., Fang X.H., Chen G. (2009). Single-molecule imaging reveals transforming growth factor-beta-induced type II receptor dimerization. Proc. Natl. Acad. Sci. USA.

[51] Zhang M., Zhang Z., He K., Wu J., Li N., Zhao R., Yuan J., Xiao H., Zhang Y., Fang X. (2018). Quantitative Characterization of the Membrane Dynamics of Newly Delivered TGF-beta Receptors by Single-Molecule Imaging. Anal. Chem..

[52] Li P., Miao Y., Dani A., Vig M. (2016). alpha-SNAP regulates dynamic, on-site assembly and calcium selectivity of Orai1 channels. Mol. Biol. Cell.

[53] Ershov D., Phan M.-S., Pylvänäinen J.W., Rigaud S.U., Le Blanc L., Charles-Orszag A., Conway J.R.W., Laine R.F., Roy N.H., Bonazzi D. (2022). TrackMate 7: Integrating state-of-the-art segmentation algorithms into tracking pipelines. Nat. Methods.

[54] Kusumi A., Sako Y., Yamamoto M. (1993). Confined lateral diffusion of membrane receptors as studied by single particle tracking (nanovid microscopy). Effects of calcium-induced differentiation in cultured epithelial cells. Biophys. J..

[55] Vega A.R., Freeman S.A., Grinstein S., Jaqaman K. (2018). Multistep Track Segmentation and Motion Classification for Transient Mobility Analysis. Biophys. J..

