# Supplementary material for: Single-Molecule Imaging Reveals Differential AT1R Stoichiometry Change in Biased Signaling
Source: Int J Mol Sci. 2023 Dec 27;25(1):374. doi: 10.3390/ijms25010374 (PMC10778740; doi:10.3390/ijms25010374)
Supplement: Supplementary file 1 [file ijms-25-00374-s001.zip › ijms-2663693-supplementary.pdf]

Single-Molecule Imaging Reveals Differential Stoichiometry Change of AT1R in Biased Signaling

Gege Qin[1,2,†], Jiachao Xu[3,†,*], Yuxin Liang[1,2], Xiaohong Fang[1,2,4,*]

[1] Key Laboratory of Molecular Nanostructure and Nanotechnology, CAS Research/Education Center for Excellence in Molecular Sciences, Institute of Chemistry, Chinese Academy of Sciences, Beijing 100190, China
[2] University of Chinese Academy of Sciences, Beijing 100049, China
[3] Key Laboratory of Molecular Developmental Biology, Institute of Genetics and Developmental Biology, Chinese Academy of Sciences, Beijing 100101, China
[4] Hangzhou Institute of Medicine (HIM), Chinese Academy of Sciences, Hangzhou 310022, China
* Correspondence: xujiachao@genetics.ac.cn (J.X.); xfang@iccas.ac.cn (X.F.)
† These authors contributed equally to this work.

KEYWORDS: single-molecule imaging, angiotensin II type 1 receptor, biased activation, stoichiometry

Figures

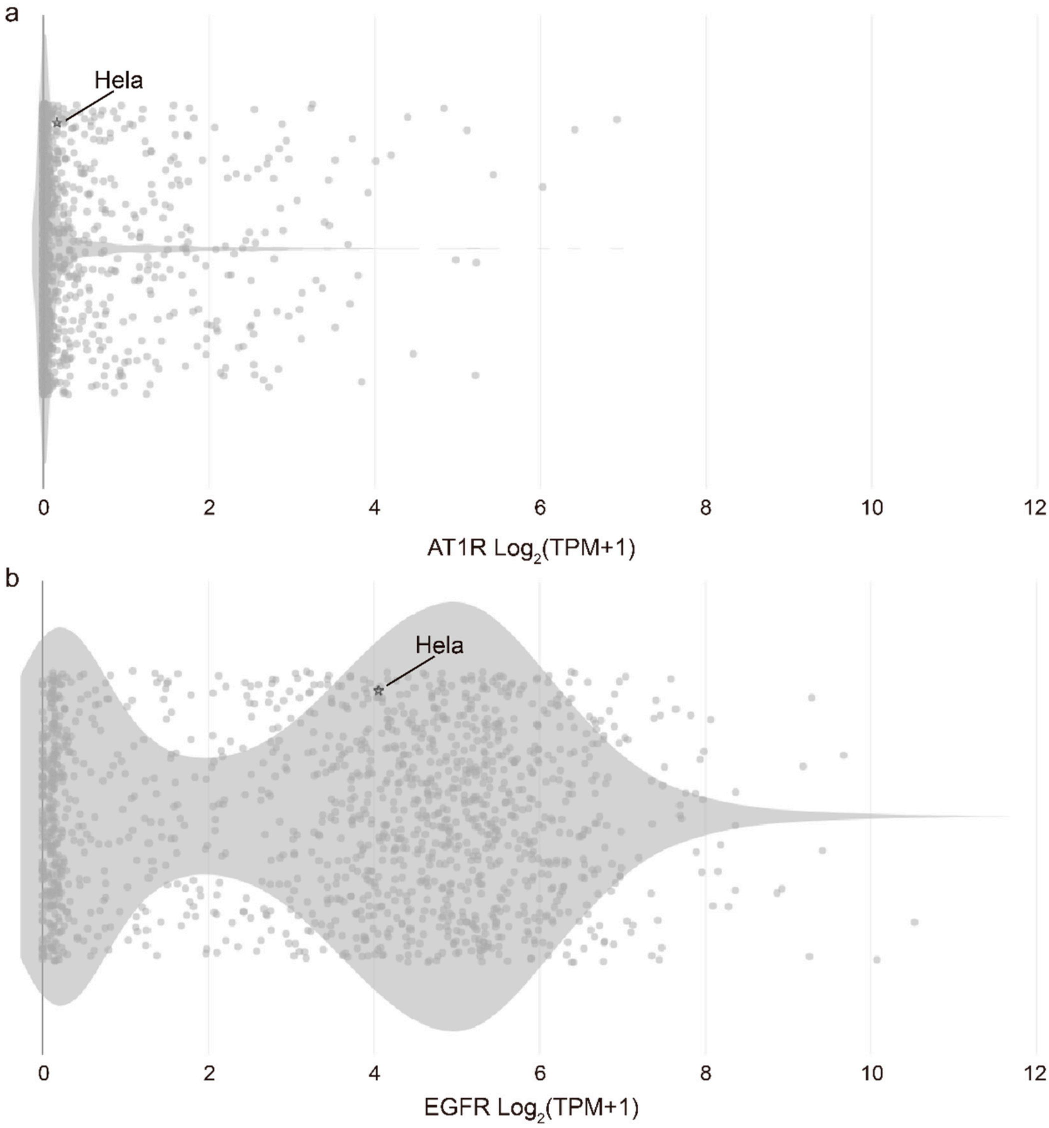

Figure S1. (a) Characterization of AT1R1 expression levels in several types of cancer cells according to DepMap expression 23Q2 Public database. (b) Characterization of EGFR expression levels in several types of cancer cells according to DepMap expression 23Q2 Public database. Comparison with EGFR, it is obvious that most cells endogenously low-express AT1R.

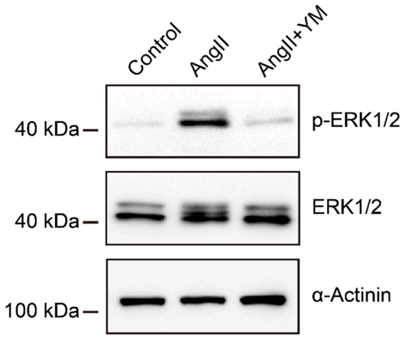

Figure S2. The effect of YM-254890 on downstream signaling of AT1R upon ligands stimulation.

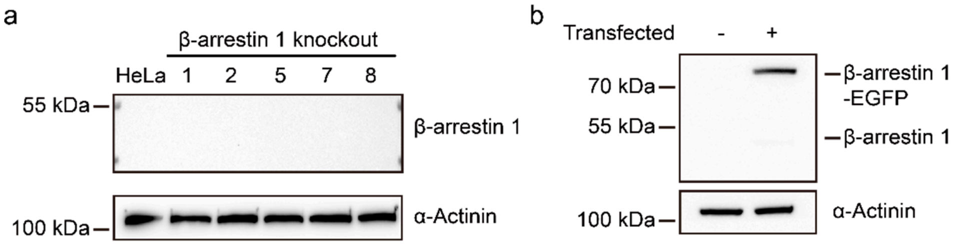

Figure S3. (a) Western blot showing the cellular expression levels of βarr1 in knock-out cell lines. (b) Western blot showing the cellular expression levels of βarr1 in Hela cells transfected with βarr1-EGFP or nontransfected, confirming the efficacy of the βarr1 antibody.

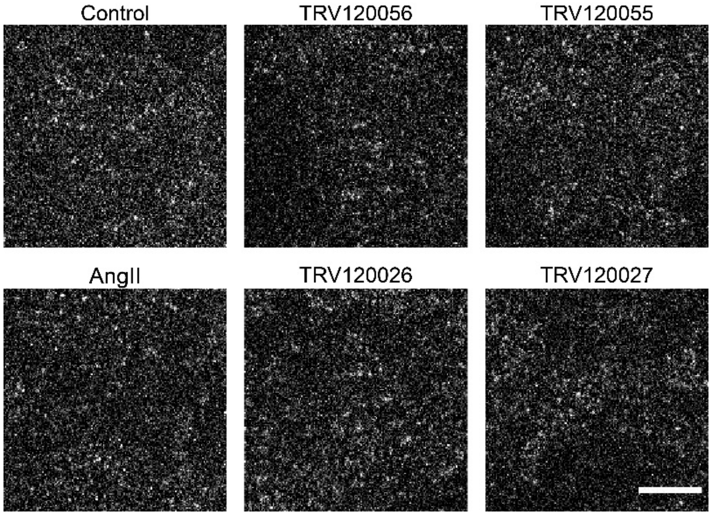

Figure S4. The representative single-molecule fluorescence images after different ligands stimulation on βarr1/2 knock-out HeLa cells. Scale bar, 10μm.

Gene	Index	Sequence (5'-3')
ARRB1	a	TTCCAGGTTGCCAGGGGTTAC<br>AACAGATTGCCACAAACTCGG
ARRB1	b	AAGGTGAAAGGGTAAGCGTGC<br>CCTCTACAGATGCCGTTGCCT
ARRB2	a	GTTGGGGTGTGTGAGGAATGA<br>CGGTGCTGAAGAGGCAGATGT
ARRB2	b	GAGGATACAGGAAAGGTACGG<br>GTCCCCAAACACAGACACATT

Figure S5. The designed primers of T7EI assay.
